# Glances at the Hospitals

**Published:** 1904-11-12

**Authors:** 


### GLANCES AT THE HOSPITALS.

### ESSEX AND COLCHESTER HOSPITAL.

The total receipts amounted to £3,630, and the expenditure to £3,186, so that the financial position of the hospital is satisfactory taken as a whole; but the committee state that the annual subscriptions have fallen by £41 lower than the previous year and a special committee has been formed to look into the matter. It is well known that this hospital is economically managed and hence is deserving of local support. Many improvements were carried out during the year. Electric light has been introduced; iron staircases and fireproof landings have replaced the old wooden ones; a lift, capable of carrying a patient on a wheeled stretcher and attendants, has been fitted up, as well as several minor alterations at a total cost of £1,636. The daily average of in-patients was 52, the cost per head per week was 24s. 10d., and the cost per bed was £12 11s. The average stay of each patient in the hospital was nearly 29 days. On January 1st the infirmary contained 59 patients, and 659 were admitted during the year, making a total of 718 under treatment. Of these 247 were discharged cured, 295 were discharged with great benefit, 30 were discharged as incurable, 65 for various reasons, and 22 died. The medical and surgical tables are very clearly arranged, and the columns show the numbers under treatment for all the diseases, the numbers "cured," "relieved," "not relieved," and "died." The operations reached a total of 291 and the result is shown in every case. An anæsthetic table is given which shows that chloroform was administered in 82 cases, gas in 16, ether in 165, A.C.E. mixture in 17, and a local anæsthetic in 7. In some instances more than one anæsthetic was used, but the above figures denote the main agent given.

